# Malaysian and Singaporean students’ affective characteristics and mathematics performance: evidence from PISA 2012

**DOI:** 10.1186/s40064-015-1358-z

**Published:** 2015-09-29

**Authors:** Lei Mee Thien, Mei Yean Ong

**Affiliations:** Research and Development Division, SEAMEO Regional Centre for Education in Science and Mathematics, 11700 Gelugor, Penang Malaysia

**Keywords:** Affective characteristics, Programme for International Student Assessment (PISA), Singapore, Malaysia, Mathematics performance

## Abstract

This paper attempts to identify the extent to which the affective characteristics of Malaysian and Singaporean students’ attainment compared to the OECD average in Programme for International Student Assessment (PISA) 2012, and examine the influence of students’ affective characteristics, gender, and their socioeconomic status on mathematics performance at both student and school levels. Sample consisted of 5197 and 5546 15-year-old Malaysian and Singaporean students. Data were analysed using hierarchical linear modelling approach with HLM 7.0 software. Results showed that the Index of economic, social, and cultural status (ESCS), mathematics self-efficacy, and mathematics anxiety have significant effects on mathematics performance in Malaysia and Singapore at the student level. Proportion of boys at the school level has no significant effects on mathematics performance for both Malaysian and Singaporean students. ESCS mean at the school level has positive and significant effects on mathematics performance in Malaysia, but not in Singapore. Limitations, implications, and future studies were discussed.

## Background

Programme for International Student Assessment (PISA) is a large-scale international assessment organised by Organisation for Economic Co-operation and Development (OECD) which measures 15-year-old students’ mathematics, science, and reading literacy every 3 years. In each cycle, one domain of literacy from mathematics, science, and reading is emphasised. The first PISA survey was conducted in 2000, followed by the cycles of 2003, 2006, 2009, and 2012. In PISA 2012, the focus was on mathematics literacy. PISA seeks to measure how young adults approaching the end of compulsory schooling are prepared in meeting challenges of the modern knowledge societies (OECD 2014). The assessment examines how these youth utilise their knowledge and skills in meeting real-life challenges, rather than mastering a specific subject or curriculum in school.

In the 2012 cycle, East Asian countries consistently outperformed the rest of the world with Shanghai, Singapore, Hong Kong, Taiwan, South Korea, Macau, and Japan being the top performing countries and economies (OECD 2014). This great achievement arose world’s attention on East Asian countries and their unique Chinese way of learning that contributed to the success (Ho 2009; Li 2004; Schneider and Lee 1990; Stevenson and Stigler 1992; Watkins and Biggs 2001; Wong 2004).

Compared to the East Asian context, Malaysia participated in PISA assessment since 2009 but obtained disappointing results in PISA 2012. Despite the push to improve the education system, there is a decline in scores and Malaysia lagged behind other Southeast Asian countries such as Singapore, Vietnam, and Thailand (OECD 2014). Malaysian students scored below the OECD average with 421 mean score in mathematics, 398 in reading, and 420 in science literacy, respectively. However, Singapore, a country neighbouring Malaysia managed to maintain as the top performer in most of the international assessments (OECD 2011). Notably, Malaysia and Singapore have similarities in ethnicity, cultures, languages, and geographical location but have huge disparity in international assessment achievements (OECD 2014).

## Overview of the education system in Malaysia and Singapore

Singapore and Malaysia have five levels of education system: pre-school education, primary education, secondary education, post-secondary education, and tertiary education with the 6 years of primary education and the 5 years of secondary education being the basic education (UNESCO 2011). According to Malaysia’s Education Act of 1996, primary education is made compulsory for children between the ages of 7+ and 12+. Students are required to sit for a national examination before going into secondary school, namely Primary School Evaluation Test (UPSR) for Malaysia and Primary School Leaving Examination (PSLE) for Singapore. PLSE determines the tracks of the students in secondary school, either normal or express track. Express track is a four-year course leading up to a Singapore-Cambridge general certificate of education ordinary-level (O-level) examination while normal track only leads up to a normal-level examination with the possibility of another year leading to O-level. In Malaysia, secondary education consists of lower and upper secondary for a period of 3 and 2 years, respectively. The assessments for lower and upper secondary of Malaysia are Form Three Assessment (PT3) and Malaysian Certificate of Education (SPM), respectively.

The mediums of teaching instruction are also different in both Singapore and Malaysia. The national language of Malaysia—Malay language is identified as the main language in teaching and learning, whereas Singapore chose the international language—English to be the teaching and learning medium though Singapore has four national languages: English, Malay, Chinese, and Tamil. It was only in 2002 Malaysia made English as the medium of instruction for science and mathematics teaching in both primary and secondary levels (Ismail and Awang 2008, 2009).

## Objective

The purpose of this study was twofold. First, examine the extent to which the affective characteristics of Malaysian and Singaporean students’ attainment in terms of their mathematics self-efficacy, mathematics self-concept, instrumental motivation, intrinsic motivation, and mathematics anxiety compared to the OECD average in PISA 2012. Second, examine the influence of students’ affective characteristics on mathematics performance after controlling student background factors in Malaysia and Singapore. Third, examine the influence of ESCS mean and proportion of boys at the school level on mathematics performance. Overall, this study attempts to address the following research questions.

1. What are the affective characteristics of Malaysian and Singaporean students that are lower or higher than the OECD average?
2. To what extent the student affective characteristics influence mathematics performance in Malaysia and Singapore?
3. To what extent the ESCS mean and proportion of boys at the school level influence mathematics performance in Malaysia and Singapore?

In this study, all the undertaken students’ affective characteristics were capitalised from this section onwards for the ease of interpretation. The gender was then aggregated and termed as proportion of boys at the school level. The terminologies of construct and variable were used interchangeably in order to avoid confusion.

## Literature review

### Mathematics self-efficacy

Bandura (1997)defined self-efficacy as belief in one’s capacity to execute the courses of action. Deriving from Bandura’s definition (1997), mathematics self-efficacy was defined as individuals’ judgements of their capabilities to solve specific mathematics problems, perform mathematics-related tasks, or succeed in mathematics-related courses (OECD 2013). For decades, self-efficacy beliefs have revealed positive relationship with academic performance (Martin and Marsh 2006; Skaalvik and Skaalvik 2004). Mathematics self-efficacy was reported as one of the stronger predictors of mathematics achievement (Stevens et al. 2006). According to Bouffard et al. (2001), students with higher level of self-efficacy have been found to be more accurate in their mathematical computations and show greater persistence on difficult items than students whose self-efficacy is low. Milford (2010) supported that students with higher self-efficacy tend to have higher academic achievement.

In PISA 2012, mathematics self-efficacy was operationalised as the extent to which students believe in their own abilities to tackle specific mathematical task. There were eight mathematics self-efficacy items with a 4-point Likert scale ranging from very confident to not at all confident in PISA 2012. The items were related to the level of confidence that students attain when dealt with the mathematics task. The sample item included “How confident do you feel about calculating how much cheaper a TV would be after a 30 % discount?”

### Mathematics self-concept

Literature review revealed that a positive association between mathematical self-concept and mathematics performance has consistently been found across different countries (Dai 2001; Marsh and Hau 2004; Marsh and Koller 2004). Students with higher level of mathematical self-concept generally show greater engagement, persistence, and effort in tasks relating to mathematics and in turn perform better than students with lower level of mathematical self-concept. More to this point, studies have also found that positive academic self-concept and self-efficacy can facilitate students’ engagement and motivation (Bong and Skaalvik 2003). In PISA 2012, mathematics self-concept was operationalised as the extent to which students perceived their competence in mathematics. There were five mathematics self-concept items with a 4-point Likert scale ranging from strongly agree to strongly disagree in PISA 2012. The items were related to the level of agreement in relation to their thinking about studying mathematics. Sample items included “I learn mathematics quickly” and “I have always believed that mathematics is one of my best subjects”.

### Motivation

Motivation was regarded as one of the driving forces for better academic performance (OECD 2013). There were two types of motivation, namely instrumental and intrinsic motivation in PISA 2012. Both constructs are central in self-determination theory (Ryan and Deci 2009) and expectancy-value theory (Wigfield and Eccles 2000). Instrumental motivation was defined as the drive to learn mathematics because students perceive it as useful to them and to their future studies and careers (Eccles and Wigfield 2002). The intrinsic motivation was represented by mathematics interest in PISA 2012. Intrinsic motivation was defined as the drive to perform an activity mainly for the joy gained from the activity itself (OECD 2013). The willingness to learn mathematics was due to the fact that they find mathematics interesting and enjoyable (Ryan and Deci 2009). As such, previous studies claimed that intrinsic motivation increased the degree of student engagement in learning and subsequently improved their performance in mathematics (e.g., d’Ailly 2003; Tavani and Losh 2003).

In PISA 2012, instrumental motivation was operationalised as students’ responses to whether they believe mathematics was important for their future career. On the other hand, intrinsic motivation indicated by mathematics interest was operationalised as students’ responses about whether they enjoy mathematics and work hard in mathematics because they enjoy the subject. The measures of instrumental motivation and intrinsic motivation in PISA 2012 database were phrased more specifically in the context of why mathematics was important. There were four instrumental motivation and intrinsic motivation items with a 4-point Likert scale ranging from strongly agree to strongly disagree in PISA 2012, respectively. The items were related to the level of agreement about students’ views on mathematics. Sample item of instrumental motivation included “Making an effort in mathematics is worth it because it will help me in the work that I want to do later on”. Meanwhile, sample item of intrinsic motivation included “I look forward to my mathematics lessons”.

### Mathematics anxiety

Mathematics anxiety was regarded as negative reaction with a feeling of tension or fear when dealing with mathematics (Richardson and Suinn 1972). Mathematics anxiety was found associated with students’ perception of their mathematics performance (Meece et al. 1990). A previous study conducted by Garry (2005) found that students who suffer from mathematics anxiety have less confidence in their ability to do mathematics (Garry 2005). The earlier study by Hembree (1990) showed that mathematics anxiety seriously constrained performance in solving mathematical tasks and reduction in anxiety is positively associated with mathematics performance. It is likely that a student’s mathematics anxiety is aroused when the student is called on to solve a mathematics problem (Ashcraft and Moore 2009). In PISA 2012, mathematics anxiety was operationalised as students’ responses to feelings of stress and helplessness when dealing with mathematics. There were five mathematics anxiety items with a 4-point Likert scale ranging from strongly agree to strongly disagree in PISA 2012. The items were related to the level of agreement in relation to their thinking about studying mathematics. Sample items included “I get very tense when I have to do mathematics homework” and “I get very nervous doing mathematics problems”.

Overall, literature revealed that students who possess higher level of mathematics self-efficacy, mathematics self-concept, instrumental motivation, intrinsic motivation, together with the lower level of mathematics anxiety would outperform those who do not possess similar levels of these affective characteristics.

### ESCS

Socioeconomic status (SES) was an individual or family’s ranking on a scale based on their ability to access or control over products or services (Mueller and Parcel 1981). Literature showed that high SES consistently has a positive impact on academic achievement regardless of the SES measures used (Sirin 2005). McConney and Perry (2010) supported that attending a school that enrols primarily students from low socioeconomic backgrounds was found to have a negative relationship with their learning outcomes. Conversely, students from families with more advantaged socioeconomic backgrounds perform better than others (Perry and McConney 2010; Stacey 2010). In fact, Sirin (2005) found that student-level SES was one of the strongest predictors of academic achievement in a meta-analysis of 74 studies examining SES and academic achievement.

Similarly, School SES composition was the aggregated measure of the social backgrounds of the students who attend a school (Milford, 2010). Increases in school SES were consistently associated with substantial increase in mathematics achievement regardless of their individual SES (Hsu 2007; Perry and McConney 2010). A substantial number of international studies involving secondary data analyses have also suggested that the association between achievement and school SES was strong for all students (e.g., Milford 2010; Shin and Slater 2010).

In PISA 2012, student-level SES was measured by the Index of economic, social, and cultural status (ESCS), a composite index of highest parental occupational status (HISEI), highest parental educational attainment (HISED), and home possessions which referred to the number of books at homes, home educational, and cultural possession (HOMEPOS). This index was derived from the students’ responses to the items related to their parents’ current jobs and educational status as well as the available number of the learning materials at home. As ESCS represented the student SES in PISA 2012; therefore, we averaged the ESCS scores of every student from a given school in calculating aggregated school-level ESCS in this study. The ESCS index was standardised in all OECD countries to have a mean of zero and a standard deviation of one. Greater values represented more advantaged family backgrounds. A negative value implies that the ESCS was below the OECD average and a positive value means that was above the OECD average.

### Gender

The influence of gender on mathematics performance is inconclusive (Friedman 1989). Some studies informed gender has no significant influence on mathematics performance (e.g., Areepattamannil and Kaur 2013; Callahan and Clements 1984). Similarly, in a large-scale assessment context, the studies using the TIMSS 2003 database indicated that there were no significant gender differences in the overall mathematics achievement across 46 participating countries at fourth and eighth grades (Mullis et al. 2004). On the other hand, previous studies have found that male students outperformed female students. For instance, Leachey and Guo (2001) found that male students have significantly higher scores than female students in the 11–13 age group. The earlier study by Ma (1995) supported male superiority in mathematics achievement. Similar results were found in PISA 2000 mathematics literacy with male students outperforming female students by 11 points across 43 participating countries (OECD 2003). However, some studies showed that female students outperformed male students (e.g., Gilleece et al. 2010; Tsai and Walberg 1983). In this study, at the student level, female and male students were recoded as 0 and 1, respectively.

## Method

### Sampling design and data sources

PISA used a two-stage stratified design sampling. First, schools having age-eligible students were sampled systematically with probabilities proportional to the school size, which is a function of the number of eligible students enrolled. Based on the selected schools, students around 15 years old were randomly selected. In this study, data used for the analysis were retrieved from the official PISA website (http://www.pisa.oecd.org). Table 1 shows the sample of demographic characteristics in Malaysia and Singapore. The total student sample were 5197 and 5546 selected from 164 and 172 selected schools in Malaysia and Singapore, respectively. The gender ratio was fairly equal.

**Table 1:** Sample’s demographic characteristics

Country	Number of schools	Female	Male
Malaysia	164	2745	2452
Singapore	172	2752	2794

### Analysis procedures

The analysis began with the comparison between the mean and standard error for the index of each construct, followed by investigation of the relationships between student and school-level variables and mathematics achievement using HLM 7.0 computer software. The HLM analysis was conducted to examine the influence of the five undertaken students’ affective characteristics on mathematics performance after controlling student background factors, namely student gender and ESCS. The multilevel model building began with a null model. The null model contains only the dependent variables, namely mathematics performance in the form of five plausible values. The null model is used to compute Intra-class correlation coefficient (ICC) by decomposing the total variance into within-school variance and between-school variance with the formulae shown in Eq. 1 (Raudenbush and Bryk 2002).

1 $${\text{ICC}} = \frac{{{\text{between school variance}}}}{{({\text{within}} - {\text{school variance}} + {\text{between}} - {\text{school variance}})}}$$

The null models served as the baseline model compared to the results of the successive models (Raudenbush and Bryk 2002). In this study, Model 1 consisted of two student background variables as control variables: gender and ESCS. Model 2 was developed by adding the five undertaken student level variables to the Model 1. Meanwhile, Model 3 was developed by adding two school-level variables, namely proportion of boys and ESCS mean to Model 2.

## Results

Notably, a positive value on an index indicates that scores obtained in a particular country were higher than the OECD average, which in turn reflects that students in this country have more positive perceptions on the undertaken affective characteristics compared to the students from other OECD countries and vice versa for an index with negative value. The interpretation is relevant to the operationalisation of each affective characteristic as discussed in the previous section. Table 2 shows that both Malaysian and Singaporean students have significant higher levels of mathematics self-concept, instrumental motivation, and intrinsic motivation compared to OECD average. However, Malaysian students were found to have lower level of mathematics self-efficacy than OECD average. In contrast, Singaporean students have higher level of mathematics self-efficacy than OECD average. On the other hand, Malaysian students have higher level of mathematics anxiety than OECD average, whereas Singaporean students have lower level of mathematics anxiety than OECD average.

**Table 2:** Indices of students’ affective characteristics of Malaysia and Singapore

Characteristics	Malaysia	Singapore
Mathematics self-efficacy	−0.25 (0.02)	0.47 (0.02)
Mathematics self-concept	0.11 (0.02)	0.22 (0.02)
Instrumental motivation	0.53 (0.02)	0.40 (0.02)
Intrinsic motivation	0.91 (0.02)	0.84 (0.02)
Mathematics anxiety	0.38 (0.03)	−0.10 (0.03)

Mean scores and standard error (parentheses) were extracted from OECD 2013 Report (Volume II). All indexes were standardised to have a mean of 0 and standard deviation of 1 across OECD countries

Based on the HLM analysis, intra-class correlation coefficient (ICC) showed that 34 and 35 % of the total variance in mathematics was attributed to schools in Malaysia and Singapore, respectively. The results indicated heterogeneity in mathematics performance among schools existing in Malaysia and Singapore. Table 3 shows that Malaysian female students tend to perform better than male students in mathematics literacy. Malaysian students from higher ESCS have also performed better than those from lower ESCS in mathematics literacy. One standard deviation increase in ESCS was associated with 16-point increase in mathematics performance. For Singapore, gender did not have significant difference between male and female students on mathematics performance. However, for Singaporean students, one standard increase in ESCS was associated with an increase of mathematics performance of about 27 points. The between-school variance explained for Malaysia was found slightly higher than Singapore with 31 % compared to Singapore of about 28 %. However, Singapore has higher within-school variance explained compared to Malaysia of about 7 and 4 %, respectively.

**Table 3:** Results of multilevel analysis

	Malaysia						Singapore					
	Model 1		Model 2		Final model		Model 1		Model 2		Final model	
	Coefficient	SE	Coefficient	SE	Coefficient	SE	Coefficient	SE	Coefficient	SE	Coefficient	SE
Intercept	415.05	4.81	414.10	4.73	417.89	3.54	563.85	4.96	567.08	3.55	566.97	3.41
Student background factors												
Boys	−3.81***	3.94	−3.39	3.57	−3.28	3.60	4.61	5.28	−3.40	4.23	−3.27	4.21
ESCS	16.03***	2.34	11.81***	2.11	7.61***	2.17	27.11***	3.13	14.28***	2.84	14.32***	2.87
School level factors												
Proportion of boys					−19.14	24.58					−3.20	21.61
ESCS mean					48.45***	7.77					−1.81	5.07
Affective characteristics												
Self-efficacy			25.14***	3.44	25.17***	3.43			36.97***	2.85	36.98***	2.86
Self-concept			−4.27	4.43	−3.14	4.46			3.30	3.78	3.23	3.70
Instrumental motivation			5.57	3.48	5.96*	3.47			−12.67*	3.41	−12.73***	3.38
Intrinsic motivation			0.10	3.69	−0.08	3.72			−6.57	3.64	−6.59*	3.64
Mathematics anxiety			−14.30***	2.65	−14.69***	2.76			−21.78***	2.97	−21.84***	2.96
Between-school variance	1422.83		1211.76		664.45		2301.01		1196.46		1192.38	
Within-school variance	3658.77		2741.94		2718.59		5824.30		4170.91		4170.18	
Total variance	5181.60		3953.70		3383.04		8125.31		5367.37		5362.56	
Between-school variance explained	31.35 %		41.54 %		67.94 %		28.12 %		62.63 %		62.75 %	
Within-school variance explained	4.20 %		28.21 %		28.82 %		7.27 %		33.59 %		33.59 %	
Total variance explained	13.75 %		32.90 %		42.58 %		14.31 %		43.40 %		43.45 %	

For Malaysia, null Model: between-school variance = 2072.73, within-school variance = 3819.21, ICC = 0.35

For Singapore, null Model: between-school variance = 3201.38, within-school variance = 6280.95, ICC = 0.34

$${\text{Proportion of variance explained at the school level }} = \tau_{00} \left( {null} \right) - \tau_{00} \left( {model 1/model2/ model 3} \right)/\tau_{00} \left( {null} \right),$$

$${\text{Proportion of variance explained at the student level }} = \sigma^{2} \left( {null} \right) - \sigma^{2} \left( {model 1/model2/ model 3} \right)/\sigma^{2} \left( {null} \right)$$

*ICC* intra-class correlation coefficient

*** *p* < .01, * *p* < .10

Model 2 shows the influence of the five selected students’ affective characteristics on mathematics performance after controlling student gender and ESCS. Mathematics self-concept, instrumental and intrinsic motivation did not have significant effects on Malaysian students’ mathematics performance in PISA 2012. The results showed that only mathematics self-efficacy and mathematics anxiety have significant effects on mathematics performance for the Malaysian model. One standard deviation increase in mathematics self-efficacy was associated with 25 points increase in mathematics performance. Meanwhile, one standard deviation increase in mathematics anxiety was associated with a decrease in mathematics performance of about 14 points. The mathematics self-efficacy and mathematics anxiety variables explained about 41 % of between-school variance and 28 % of within-school variance.

Compared to the Malaysian model, Singaporean students’ mathematics self-efficacy and mathematics anxiety have relatively higher effects on mathematics performance. One standard deviation increase in mathematics self-efficacy was associated with 37 points increase in mathematics performance, whereas one standard deviation increase in mathematics anxiety was associated with 22 points decrease in mathematics performance. In addition, instrumental motivation had negative and significant effects on mathematics performance. One standard deviation increase in instrumental motivation was associated with a decrease in mathematics performance of about 13 points. These three student-level variables contributed about 63 and 34 % of between-school variance and within-school variance, respectively.

The final model showed the influence of two school level variables, namely ESCS mean and proportion of boys after controlling the student background factors and the selected students’ affective characteristics. For the final Malaysian model, mathematics self-efficacy and mathematics anxiety remained having significant effects on mathematics performance. ESCS mean at the school level had positive and significant effect on mathematics performance with one standard deviation increase in ESCS mean associated with an increase in mathematics performance of about 48 points. Proportion of boys at the school level did not have significant effect on mathematics performance. The final model explained about 68 and 29 % of between-school and within-school variance, respectively.

For the final Singaporean model, mathematics self-efficacy, instrumental motivation, and mathematics anxiety remained having significant effects on mathematics performance. Intrinsic motivation had negative and significant effect on mathematics performance with one standard deviation increase in intrinsic motivation associated with a decrease in mathematics performance of about 22 points. Different from the Malaysian final model, ESCS mean at the school level did not have significant effect on mathematics performance in the final Singaporean model. As such, it is expected that the between-school variance and within-school variance in Model 3 were not much different from Model 2. The final Singaporean model explained about 63 and 34 % of between-school and within-school variance, respectively. Overall, both the final Malaysian and Singaporean models contributed about 43 % of total variance explained on mathematics performance.

## Discussion and conclusion

Despite certain similarities in social cultural context and geographical location, the wide performance gap between Malaysia and Singapore in PISA 2012 has prompted this study to examine the mathematics performance in PISA between these two countries. The findings reflected that both Malaysian and Singapore students enjoyed learning mathematics and students believe that mathematics is important for their future careers in addition to perceive their mathematics competency positively. The findings supported the previous studies (e.g., d’Ailly 2003; Tavani and Losh 2003) with the positive relationship between motivation and mathematics performance. The current findings with the positive students’ perception of their competence in mathematics in both Malaysia and Singapore have also been found consistent with the studies conducted by Hembree (1990) and Garry (2005).

However, Singaporean students have higher ability to solve a range of pure and applied mathematics problems, hence explain the outstanding mathematics performance compared to Malaysian students with the relative lower level of mathematics self-efficacy. For Singaporean students, their outstanding performance in mathematics literacy could be further explained by their lesser feelings of stress and helplessness when dealing with mathematics. In this regard, higher levels of anxiety and stress in learning mathematics among the Malaysian students could be related to their underperformed results. The findings further supported that “Mathematics anxiety is a significant impediment to mathematics achievement” as claimed by Ashcraft and Moore (2009).

In a related vein, it is important to highlight that medium of instruction has impact on students’ mathematics, performance (Klein et al. 2013; Launio 2015). Since 1970s, the medium of instruction in the primary and secondary mathematics subject is English language in Singapore until present (Woo, public communication, August 28, 2015). Unlike Singapore, the national language in Malaysia is Malay language. Malay language is the medium of instruction in the mathematics subject until 2002. Therefore, one of the possible reasons to explain the higher level of mathematics anxiety of Malaysian students could be due to the changes of medium of instruction, especially during the transition period of the implementation of English language as the medium of instruction in all government schools except international schools since 2002. A declining performance in both subjects in the Trends in International Mathematics and Science Study (TIMSS) 2011 could be another evidence to support that mathematics anxiety does exist among the Malaysian students due to the language change in the mathematics subject. The medium of instruction in mathematics and science subjects was changed back to Malay language in 2012 in line with a new education policy.

Another reason to explain the disparity of mathematics performance in PISA 2012 between Malaysia and Singapore could be due to the different levels of mathematics self-efficacy that students attained. The HLM findings of this study have supported this point of view based on the positive relationship between mathematics self-efficacy and mathematics performance in both countries.

Singaporean students have revealed different results from the literature with the lesser the Instrumental and intrinsic motivation, the better the mathematics performance they might have. Such ‘odd’ results could be associated with the issue of suppression effect between the student-level variables in hierarchical linear modelling (Darmawan and Keeves 2006). Theoretical details of suppression effects could be referred to Darmawan and Keeves (2006) as well as Krus and Wilkinson (1986). In this study, it is likely that instrumental motivation is highly correlated with intrinsic motivation. The effect of instrument motivation on mathematics performance might suppress the effect of intrinsic motivation on mathematics performance. In addition, the complexity of mediating or moderating process between motivation and students’ performance in mathematics literacy that involves possible moderators and mediators such as parental background and school environment could be further investigated with the insiders using qualitative approach (Coon et al. 1993; Tian 2006).

Schools with higher or lower proportion of boys did not seem to influence Malaysian and Singaporean students’ performance in mathematics. Despite the findings could be due to a well-agreed-on reason that gender differences are contextualised and vary across mathematics domains (Hsu 2007), at least, the current findings inform the possible gender equality in mathematics performance in Malaysia and Singapore. The diversity in student socioeconomic background is considered as an influential factor of mathematics performance within-school in both Malaysia and Singapore. In this regard, parental education status and family socioeconomic background could contribute to explain their children performance in mathematics indirectly. The higher the level of parental education status and family socioeconomic background, the better the performance students attain. Different from Singapore, education opportunity with relatively high quality and equity seems not equally distributed for students from different socioeconomic backgrounds between Malaysian schools. Such findings are understandable as it is not easy to implement the education policies in Malaysia with a land area around 330 times larger and a population of six times more than Singapore (Jones 2013). The difficulties of educational policies’ implementation are much higher compared to Singapore.

The findings of this study could be used to inform better classroom teaching practices. Teachers could modify or improve their instructional strategies with trainings and efforts to increase students’ self-efficacy and motivation, hence reduce the level of anxiety in learning mathematics. This is because teachers’ support is found preferring to display positive forms of motivation and academic accomplishments (Wentzel et al. 2010). For policy implications, the findings further provide a possibility to reinvestigate the common practices of streaming the schools based on student academic performance in order to reduce the performance gaps between schools.

However, the findings’ interpretations served no causation because PISA 2012 data were cross-sectional in nature. This study was also limited to the student background questionnaire of affective characteristics in PISA 2012. The items of students’ affective characteristics might be too general and therefore might not able to fully capture their perception of affective characteristics. In addition, it is rather difficult to have the content of each item being perceived and interpreted in the same way across groups from different contexts and cultural background (Täht and Must 2013). In relation to this, measurement invariance approach could be employed to identify the items that convey the same meaning across groups prior to conduct the analyses between countries (Byrne 2008). With respect to this concern, from a methodology perspective, measurement invariance across Malaysian and Singapore PISA dataset should be examined prior to further analysis to ensure a comparable and rigorous findings could be produced in future studies.

As pointed out by Hattie (2009), students’ mathematics performance does not depend on their affective characteristic per se, but other factors such as teaching approaches have also contributed to a large proportion of unexplained variance.

The current findings could be cross-validated using data in the next cycles of PISA. Future studies can be conducted by using different methods such as classroom observation and interview to get deeper understanding of how students’ socioeconomic background and their affective characteristics influence mathematics performance. It is worth to highlight that not all effects on students’ outcomes are direct (Klieme 2013). Overall, this study contributes to inform student socioeconomic status and their affective characteristics remain firmly entrenched as an important aspect of the schooling outcome, which concurrently informs education indicators at the system level.
